# Editorial: Inflammation, aging, and disease: new perspectives and interventions

**DOI:** 10.3389/fragi.2023.1228756

**Published:** 2023-06-26

**Authors:** Niharika Arora Duggal, Sian M. Henson, James E. Turner

**Affiliations:** ^1^ Institute of Inflammation and Ageing, College of Medical and Dental Sciences, University of Birmingham, Birmingham, United Kingdom; ^2^ William Harvey Research Institute, Barts and the London School of Medicine and Dentistry, Queen Mary University of London, London, United Kingdom; ^3^ School of Sport, Exercise and Rehabilitation Sciences, College of Life and Environmental Sciences, University of Birmingham, Birmingham, United Kingdom

**Keywords:** inflammation, immunosenescence, ageing, gerontology, disease

Population aging is accelerating worldwide. In 2019, there were 703 million people aged 65 years or older in the world, and this number is projected to double to 1.5 billion people by 2050. Between 2009 and 2019, the global proportion of people aged 65 years or over increased from 6% to 9% and is projected to rise further to 16% by 2050. Globally, a person aged 65 years in 2015–2020 is expected to live for an average of 17 years, but this number will have increased to 19 years between 2045 and 2050 (United Nations Department of Economic and Social Affairs, 2019). A global challenge is to identify factors that influence healthy ageing and develop strategies to limit age-associated disease. Thus, research that improves understanding of aging mechanisms is essential.

This Research Topic includes four articles: a review, a mini review, an original investigation, and a perspective. Two of the articles, a review by Schmitz et al. and a mini-review by Cao provide a mechanistic link between age-associated inflammation and DNA damage. The original investigation by Mao et al. examines associations between C-Reactive Protein (CRP) or lifestyle with polymorphisms in genes that counter oxidative stress or DNA damage. Finally, the perspective by Soloski et al. hypothesises that older adults living in Sardinia—an Italian Island in the Mediterranean sea—exhibit exceptional longevity due to a unique pattern of pathogen exposure combined with lifestyle and subsequent effects on the immune system.

Aging is characterised by an increase in systemic markers of inflammation (i.e., inflammaging) and changes to the phenotype and function of most immune cells, driving impaired immunity against infections and possibly malignancies (i.e., immunosenescence) (Pawelec et al, 2020; Fulop et al, 2023). However, this interpretation is nuanced: the two processes are interconnected, some age-associated changes may be beneficial (Fulop et al, 2018), and many questions remain unanswered. For example, although a variety of cells and tissues contribute to the age-associated increase in inflammation, not all molecular mechanisms underlying aberrant cytokine production are fully understood. Given that DNA damage accumulates in senescent cells, inducing a pro-inflammatory senescence-associated secretory phenotype, Schmitz et al. implicate the cyclic guanosine monophosphate-adenosine monophosphate (cGAMP) synthase (cGAS) and the adaptor stimulator of interferon genes (STING) pathway as an important link between senescence, inflammation and immunity. Upon detection of single or double strand DNA breaks in the cytoplasm, the cGAS-STING pathway leads to type I interferon production. The pathways is implicated in inflammatory disease and has been shown to be essential for monocyte, CD4^+^ and CD8^+^ T cell function. In support, the mini-review by Cao implicates the cGAS-STING pathway in the aberrant production of the type I interferons in the absence of pathogenic infection. Aligned with the “transposon theory of ageing,” Cao proposes that type I interferons drive inflammaging in response to an accumulation of transposable elements with viral reminiscence, which account for around half of the human genome (Bourque et al, 2018; Klein and O’Neill, 2018). In support of this hypothesis, Cao summarises several studies undertaking transcriptomic, epigenomic and proteomic analyses across a variety of animal tissues, showing an upregulation of interferon-response genes, or genes inducible by interferon, with ageing.

Given the role of reactive oxygen species in inflammatory disease and in DNA damage, Mao et al. genotyped 22 single nucleotide polymorphisms (SNPs) within 3 antioxidant enzyme genes and 79 SNPs within 14 DNA base excision repair genes among 333 adults age 30–74 years. Correlations between the SNPs and CRP were examined, leading to computation of genetic risk scores for antioxidant enzyme genes (*CAT and MnSoD*) and base-excision repair genes (*MUTYH*, *SMUG1*, *TDG*, *UNG*, and *XRCC1*). Comparing people with the highest genetic risk scores to the lowest, CRP concentration was 13.9% higher (*p* = 0.30) in the antioxidant gene analysis, and 57.4% higher (*p* = 0.009) in the base-excision repair gene analysis. Thus, Mao et al. provide the first evidence of a positive association between a base-excision repair genetic risk score with inflammation. In addition, using a validated self-report questionnaire assessing alcohol intake and physical activity, in combination with measured BMI (Byrd et al, 2019), participants with higher vs. lower scores (interpreted as a “proinflammatory” vs. “anti-inflammatory” lifestyle respectively) had 120% higher CRP (*p* < 0.001) but there were no statistically significant interactions between lifestyle and the genetic risk scores. Future research, measuring aspects of lifestyle more precisely (e.g., physical activity behaviours assessed using accelerometry, body composition assessed using dual energy x-ray absorptiometry) might reveal stronger interactions with antioxidant defences and DNA repair processes.

In the perspective by Soloski et al. the influence of lifestyle, in combination with a unique past exposure to infectious disease, is hypothesised as explaining exceptional longevity among older adults living in the so-called “blue zone” of Sardinia. Several studies are summarised in the perspective that have compared residents of villages in Sardinia with high and low longevity, implicating multiple factors linked to successful ageing, including a physically active lifestyle (e.g., farming—shepherding), lower body mass index, living at altitude, and effective stress coping mechanisms. However, Soloski et al. explain that people living in this region in the early 1900s, when compared to other regions, or people living in a similar region after the 1950s, had a different pattern of exposure to *Helicobacter pylori*, helminth infections, malaria, tuberculosis, and enteric infectious disease. Indeed, Soloski et al. hypothesised that this pattern of exposure to infections has led to “trained immunity” (also known as “innate immune memory”) influencing longevity via an anti-inflammatory “fingerprint”. For example, it has been shown that helminth infections do not induce a classical pro-inflammatory response, and instead are characterised by type 2 cytokine production (i.e., IL-4, IL-5, IL-9, and IL-13) (Ludwig-Portugall and Layland, 2012). In addition, *H. pylori* elicits a weak immune response, leading to persistent infection, regulatory T cell development, and phagocyte reprogramming to produce the anti-inflammatory cytokine IL-10 (Kaebisch et al, 2014; Altobelli et al, 2019). Thus, older adults living in this region, could provide an important population for further study and comparison to other cohorts.

In summary, this Research Topic has brought to light novel insight into underlying mechanisms driving inflammaging and has highlighted new areas for research that could lead to interventions for promoting successful and healthy aging.

## References

[B1] Altobelli A. Bauer M. Velez K. Cover T. L. Müller A. (2019). *Helicobacter pylori* VacA targets myeloid cells in the gastric lamina propria to promote peripherally induced regulatory T-cell differentiation and persistent infection. mBio 10 (2), 002611-19–e319. 10.1128/mBio.00261-19 PMC6426600 30890606

[B2] Bourque G. Burns K. H. Gehring M. Gorbunova V. Seluanov A. Hammell M. (2018). Ten things you should know about transposable elements. Genome Biol. 19 (1), 199. PMID: 30454069; PMCID: PMC6240941. 10.1186/s13059-018-1577-z 30454069 PMC6240941

[B3] Byrd D. A. Judd S. E. Flanders W. D. Hartman T. J. Fedirko V. Bostick R. M. (2019). Development and validation of novel dietary and lifestyle inflammation scores. J. Nutr. 149 (12), 2206–2218. 10.1093/jn/nxz165 31373368 PMC6887697

[B4] Fulop T. Larbi A. Dupuis G. Le Page A. Frost E. H. Cohen A. A. (2018). Immunosenescence and inflamm-aging as two sides of the same coin: Friends or foes? Front. Immunol. 8, 1960. PMID: 29375577; PMCID: PMC5767595. 10.3389/fimmu.2017.01960 29375577 PMC5767595

[B5] Fulop T. Larbi A. Pawelec G. Khalil A. Cohen A. A. Hirokawa K. (2023). Immunology of aging: The birth of inflammaging. Clin. Rev. Allergy Immunol. 64 (2), 109–122. Epub 2021 Sep 18. PMID: 34536213;. 10.1007/s12016-021-08899-6 34536213 PMC8449217

[B6] Kaebisch R. Mejías-Luque R. Prinz C. Gerhard M. (2014). *Helicobacter pylori* cytotoxin-associated gene A impairs human dendritic cell maturation and function through IL-10-mediated activation of STAT3. J. Immunol. 192 (1), 316–323. Epub 2013 Nov 29. 10.4049/jimmunol.1302476 24293633

[B7] Klein S. J. O’Neill R. J. (2018). Transposable elements: Genome innovation, chromosome diversity, and centromere conflict. Chromosome Res. 26 (1-2), 5–23. Epub 2018 Jan 13. 10.1007/s10577-017-9569-5 29332159 PMC5857280

[B8] Ludwig-Portugall I. Layland L. E. (2012). TLRs, treg, and B cells, an interplay of regulation during helminth infection. Front. Immunol. 3, 8. 10.3389/fimmu.2012.00008 22566894 PMC3342019

[B9] Pawelec G. Bronikowski A. Cunnane S. C. Ferrucci L. Franceschi C. Fülöp T. (2020). The conundrum of human immune system "senescence. Mech. Ageing Dev. 192, 111357. 10.1016/j.mad.2020.111357 32949594 PMC7494491

[B10] United Nations Department of Economic and Social Affairs. 2019 World population ageing 2019 highlights. Available at: https://www.un.org/en/development/desa/population/publications/pdf/ageing/WorldPopulationAgeing2019-Highlights.pdf (accessed May 04, 23).

